# A Review on 3D-Printed Templates for Precontouring Fixation Plates in Orthopedic Surgery

**DOI:** 10.3390/jcm9092908

**Published:** 2020-09-09

**Authors:** Rodica Marinescu, Diana Popescu, Dan Laptoiu

**Affiliations:** 1 Department of Orthopedics, University of Medicine and Pharmacy Carol Davila, 020021 Bucharest, Romania; rodicamarinescu@ymail.com; 2 Department of Robotics and Production Systems, University Politehnica of Bucharest, 060042 Bucharest, Romania; 3 Department of Orthopedics 2, Colentina Clinical Hospital, 020125 Bucharest, Romania; danlaptoiu@yahoo.com

**Keywords:** orthopedic surgery, fixation plate, precontouring, 3D printing, fractures

## Abstract

This paper is a systematic review of the literature on 3D-printed anatomical replicas used as templates for precontouring the fixation plates in orthopedic surgery. Embase, PubMed, Cochrane, Scopus and Springer databases were consulted for information on design study, fracture anatomical location, number of patients, surgical technique, virtual modeling approach and 3D printing process. The initial search provided a total of 496 records. After removing the duplicates, the title and abstract screening, and applying exclusion criteria and citations searching, 30 papers were declared eligible and included in the final synthesis. Seven studies were identified as focusing on retrospective non-randomized series of clinical cases, while two papers presented randomized case control studies. Two main approaches were highlighted in developing 3D-printed anatomical models for precontouring fixation plates: (a.) medical reconstruction, virtual planning and fracture reduction followed by 3D printing the model; (b.) medical reconstruction followed by 3D printing the model of the mirrored uninjured side. Revised studies reported advantages such as surgical time and blood loss reduction, while the reduction quality is similar with that of the conventional surgery. During the last couple of years there was an increase in the number of studies focused on precontouring orthopedic plates using 3D printing technology. Three-dimensionally-printed templates for plate precontouring were mostly used for acetabular fractures. Knowledge on medical virtual modeling and reconstruction is mandatory.

## 1. Introduction

In 3D printing (3DP) technology (also known as rapid prototyping (RP) or additive manufacturing (AM)) the development efforts are nowadays mostly focused on new applications and materials, as well as on enhancing the hardware and dedicated software performances [1]. Following this trend, the medical domain benefits from 3DP, which better responds to the requirements of personalization (one of the keys in improving healthcare), availability and affordability than the traditional manufacturing processes [2]. Subtractive and forming technologies are more suitable for mass production than for prototyping, which requires expensive equipment for the industrial environment, as well as specific tools, fixtures and operators’ skills [3]. On the contrary, 3DP technology can be made available in hospitals and universities, because operating the equipment is a simpler task.

Different literature reviews have revealed an increasing use of 3D-printed models in surgery [4], orthopedics [5] and orthopedic trauma [6], interventional radiology [7], surgical teaching and assessment [8], etc. The reported advantages of 3DP-based approaches refer to the strong capabilities of customization based on patient imagistic data (computer tomography (CT), magnetic resonance imaging (MRI)), improved visualization of anatomy allowing better diagnosis evaluation, a decrease in operating time and radiologic exposure during surgery, improved intervention accuracy, and enhanced communication among physicians and with patients [4,6].

3D prints can be used as custom implants [9] and as devices for increasing surgical accuracy (patient-specific surgical guides) [10], but currently their main purpose is to serve as anatomical replicas [4,11]. In orthopedic surgery, tangible 1:1 models of patient anatomy allow better visualization, which is very important for evaluating complex fracture patterns. 3D-printed bone replicas are also helpful in measuring screw lengths, in choosing the fixation plates or implants, and precontouring (pre-shaping) the fixation plates [6,12].

There are many reviews addressing the applications of 3DP technology in medicine, and in particular for orthopedic surgery [4,5,6,10,12]. However, a detailed analysis of the use of 3D-printed models for precontouring orthopedics fixation plates has not been performed so far, to the authors’ knowledge. Thus, for raising awareness on the particularities and usefulness of this type of application, and offering the basic knowledge for those willing to develop and use such 3D-printed models in clinical work, a systematic literature review was conducted in this paper. The interest in a deeper study on this topic also resides in the particularity of these models to be both a 1:1 replica of the patient bone anatomy and a medical device to be used pre- and intra-operative. This implies specific development approaches that were identified and discussed. The favorite type of interventions for which 3D-printed templates were used, the clinical evidences of their effectiveness, and their advantages and limitations were also presented.

The research questions (RQ), and corresponding objectives (Obj) aimed at by this review were the following:

− RQ1: What is the reported use of 3D-printed models for precontouring plates in orthopedic surgery? → Obj1. Presents a state of the art reference document through performing a systematic review.
− RQ2: What are the approaches in developing and using 3D-printed models for precontouring plates? → Obj2. Identify the typical workflows currently used in this field.
− RQ3: What are the reported advantages and challenges? → Obj3. Discuss the review results, reported benefits and shortcomings, and preferred anatomical zones for this type of application.

## 2. Materials and Methods

The systematic review was conducted in accordance with the preferred reporting items for systematic reviews and meta-analyses (PRISMA) guidelines. No ethics approvals were required for this research.

### 2.1. Search Strategy

The systematic literature review was performed on PubMed, Embase, Scopus, Springer and Cochrane databases. The set of search keywords referred to the manufacturing process (3D printing or 3D-printed or rapid prototyping or additive manufacturing), the device of interest (plate) and the type of operation performed on the plate (precontouring, pre-bending, pre-shaped, pre-bent). An interesting observation was that the term “additive manufacturing” (the standardized name of the technology) is usually used in the studies on metallic implants or custom designed plates using specific metal based processes such as EBM (electron beam melting) or SLM (selective laser melting). These processes are not used for medical applications in which plastic models suffice, as is the case for the topic reviewed in this article. An initial questioning based on a combination of all these three categories of keywords provided very few results. Therefore, in order to avoid excluding relevant papers, the next search used only the word “plate” and three alternative names for the manufacturing technology. The optimized search terms are detailed bellow:

Pubmed: (((3d printing [Title/Abstract] OR 3d-printed [Title/Abstract] OR rapid prototyping [Title/Abstract]) AND plate [Title/Abstract]) AND English [Language]) AND (“2001” ([Date-Publication]: “2019” [Date-Publication]), Activated filter: humans;

Embase: (‘3d printing’: ti, ab, kw OR’ 3d printed’: ti, ab, kw OR ‘rapid prototyping’: ti, ab, kw) AND plate: ti, ab, kw AND English: la AND [2001–2019]/py, activated filters: study types, journal titles;

Cochrane: 3d printing or 3d-printed or rapid prototyping in Title Abstract Keyword AND plate in Title Abstract Keyword-with Cochrane Library publication date Between January 2001 and September 2019;

Scopus: TITLE-ABS-KEY (“3d printing” OR “3d-printed” OR “rapid prototyping”) AND TITLE-ABS-KEY (plate) AND LANGUAGE (English) AND PUBYEAR > 2001 AND (LIMIT-TO (SUBJAREA, “MEDI”)) AND (LIMIT-TO(DOCTYPE, “ar”)) OR LIMIT-TO (DOCTYPE, “cp”));

Springer link: plate AND (“3d AND printing” OR “rapid AND prototyping” OR “3d-printed”)’within English, additional filter: Medicine and Public Health, Orthopedics, 2001–2019.

### 2.2. Study Selection

Data was collected using three modalities: electronic databases tracking based on keywords, tracking based on the references of full-text screened studies and tracking based on citations of full-text screened studies. The time frame for literature search was January 2001 to November 2019. Only studies in English were considered. Included studies fulfilled the following criteria: investigating humans, not referring to patient-specific surgical guides or metal 3DP/RP/AM customized surgical plates, containing information of the medical modeling and the templates 3DP related aspects.

### 2.3. Data Extraction

Two authors independently performed the search based on the aforementioned keywords. Duplicated studies were removed using an excel spreadsheet. Title and abstract screening for identifying eligible studies was also made independently by two authors. Five additional papers on the topic were found within the references of screened papers and among their citations. Despite the general search expressions, there were several studies identified by snowballing. These contained, for instance, the term “stereolithography” in the title and abstract and not “3D Printing”, or “preoperative planning” and “acetabular fracture” for describing the 3DP application. By paying careful consideration to the references from the revised literature, the potential situation of missing a relevant study was diminished. Information from the full-text screened studies was synthesized in data-extraction forms containing the following items: design study, type of intervention, surgical technique, level of evidence, number of patients, approach and descriptive information on virtual modeling and 3DP process. The completed forms were discussed by authors for reaching agreement. Six studies with insufficient or non-relevant data for the review were removed after full text reading. One paper was a duplicated dissemination of a case report, in three papers the abstracts did not clearly reflect the papers’ content, in one paper data on patients was missing, while another paper lacked the basic information considered for this review.

### 2.4. Data Analysis

Considering the different levels of evidence of reviewed studies and their corresponding modalities to report outcomes, a narrative synthesis was carried out. Templating in different body regions, surgical techniques, as well reported advantages were extensively discussed. Aspects directly related to the 3DP process and to the virtual medical modeling were also included in the data extracted from the reviewed studies.

Data gathered from the literature review have revealed a division of studies on the following categories: case reports, series of cases, randomized and non-randomized clinical studies. It also showed a preference for using 3D-printed precontouring templates for acetabulum fractures more than for clavicle, ribs or calcaneus fractures. Two approaches in developing this 3DP-based medical application were identified: the most complex one involving virtual simulation being less used than the other approach, where 3D-printed templates of the injured zone or mirror of the unaffected zone were virtually reconstructed and manufactured.

Data was analyzed and discussed in order to answer the research questions and meet the review objectives. As a consequence, data was synthetized in a general table from which the following were extracted and contextually discussed: reported use of 3D-printed models for precontouring (RG1), main development approaches (RG2), advantages and challenges (RG3).

## 3. Results

**Figure 1:**
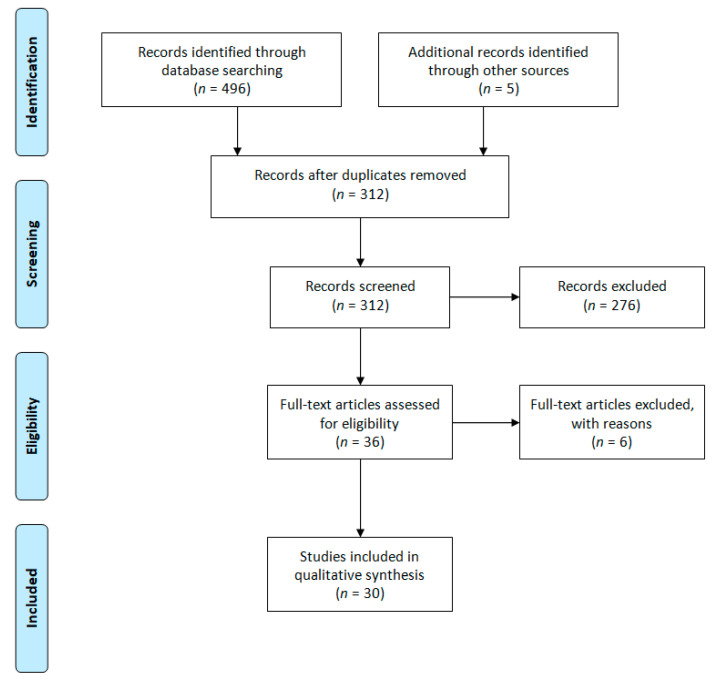
Searching strategy (PRISMA).

Figure 1 shows the results of the search strategy and selection criteria. The initial search provided a total of 496 records. After performing duplicates removal, title and abstract screening, applying exclusion criteria and searching for references and citations, as described in the method section, a group of 30 papers was considered eligible and included in the final synthesis.

Table 1 presents an overview of the reviewed studies based on the data-extraction form, answering RQ1 in a synthetic manner. The data included in further tables and Figures are also building the current perspective over the field as aimed at by Obj1.

The studies included in the review were conducted by researchers from 12 countries (e.g., China—14 studies (46.6%), South Korea—4 studies (13.3%), the two randomized case-controlled studies being carried out in India).

### 3.1. Studies Design

The systematic review showed the predominance of case reports (11 papers, representing 36.67% of all included studies) and series of cases (ten papers, 33.33% of all included studies). Case reports were included in this review as they provided valuable information on how 3D-printed templates for plate precontouring were obtained and used in practice for different types of interventions.

Seven papers, i.e., 23.33% of all included studies, are retrospective non-randomized series of cases [19,20,23,24,28,29,38]. In these papers, 3D prints were used as templates for plate precontouring for 128 patients out of 233 patients surgically treated.

Randomized case-controlled studies are presented in two papers [30,31], representing 6.67% from all reviewed studies. The total number of patients included in randomized case-controlled studies was 46 from which 22 were treated using precontoured fixation plates and 24 were part of the control group. In [30] the clinical studies included patients from June 2012 to December 2014, while in [31] the period was 1 October 2014 to 1 March 2016.

In all reviewed studies, 303 patients with different diagnosis were treated using 3D-printed models. The majority of studies are focused on fractures, only in two cases non-union [35] and corrective osteotomies [26] were discussed.

Table 2 is a synthesis of the descriptive text presented above regarding the study design and number of patients for whom 3D-printed models were manufactured and used as templates for plate contouring before surgery.

### 3.2. Studies Quality

The MINORS (Methodological Index for Non-Randomized Studies) scale was used for assessing the methodological quality for the comparative. Two studies were of level II of evidence [30,31], five of level III [20,23,24,28,29], most case series (thirteen) were of level IV and the remaining ten were of level V.

### 3.3. Anatomical Locations of Reported Cases

The following anatomical locations were reported in the reviewed studies (Table 3): acetabulum (and pelvis); clavicle (and os acromiale); ribs; scaphoid; calcaneus; humerus; cubitus.

### 3.4. Data on Reported Advantages

Table 4 synthetized the data extracted from nine reviewed studies (non-randomized and randomized ones) presenting details on blood loss, surgery time and quality of reduction, thus responding to RG3. Instrumentation times are also noted where available.

Battiato et al. report their experience by comparing surgery with and without 3D prints [14]: 45 min reduction in surgery time, 1 min of fluoroscopy instead of 2 min for the classical procedure and 500 mL blood loss in comparison with 1000 mL, mentioning that the use of 3D prints is beneficial for complex and not for simple fractures. In [30], the authors also note blood loss reduction (mean 620 vs. 720 mL in classic surgery) and 12 min less in surgery time, these however not being considered significant. Additionally, Li at al. [29] noted that the blood loss decrease was not significant for the 3DP group, but the mean operation time was 43 min shorter. Hung et al. reported 57 min reduction in instrumentation time for the 3D prints group [24].

In their long-term retrospective review of clinical cases, Li et al. [28] noted significant less intraoperative blood loss (481.4 ± 103.2 mL vs. 771.1 ± 114.4 mL), blood transfusion and operation time (128.9 ± 59.2 min vs. 191.4 ± 85.1 min) for 3DP experimental group in comparison with the control group. They also comparatively assessed the healing time, complication rates, Matta and Majeed scores. All these criteria were favored by the use of pre-contouring plates in treating tile C pelvic fractures.

A comparative study of plate precontouring using Synbones (synthetic bone models used for training or surgery simulation purposes) and 3D-printed patient-specific anatomical models is presented in [38]. Again, the surgery duration was smaller for the 3D prints group (18 min less) and 15 mL less blood loss. This was explained by the fact that for Synbone group, surgeons had to supplementary adjust the plates during surgery as their patients’ humerus were shorter that the standard Synbone model.

Chen et al. [20] also reported blood loss reduction (696.07 ± 166.54 mL vs. 833.75 ± 227.44 mL) and surgery time decrease (157.5 ± 20.48 min vs. 157.5 ± 20.48 min), but no statistically significant differences in reduction quality (Matta score) of scoring of hip function scoring (Merle d’Aubigné score).

### 3.5. 3D Printing Based Approaches

A typical workflow for all medical applications assisted by 3DP technology is based on the use of patient CT scanning data for virtually reconstructing bone anatomy and then 3D printing the injured and/or uninjured zone [43]. This information specifically answers RG2 and Obj2, but also to RG1 and Obj1 by documenting the state-of-the-art in relation to the processes of virtual medical reconstruction, precontouring templates development and 3DP.

**Figure 2:**
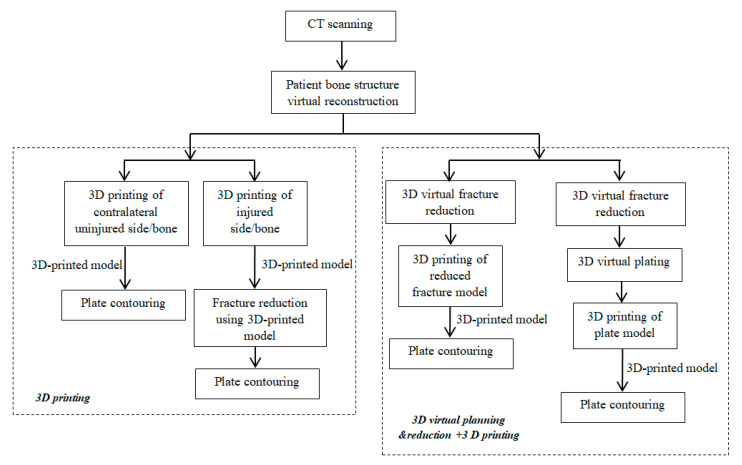
3D prints development flow showing the approaches used in included studies.

The studies included in the qualitative synthesis identified two main approaches in developing and using 3D prints for precontouring fixation plates. These are illustrated in Figure 2. One approach uses only the 3D-printed model (of the injured side or of the mirrored uninjured side) as template for plate precontouring, while the other approach uses virtual planning and reduction simulation for generating a virtually reduced fractured model, followed by 3D printing this model for using it as a template for plate precontouring.

3D-printed model-based approach: Virtual model of intact bone/zone is reconstructed by mirroring and it is 3D-printed. This 3D-printed replica is used for pre-contouring the plate by assuming body symmetry [16,17,18,20,21,22,25,27,28,35,36,37,38,39,40]; Virtual model of injured bone/zone is reconstructed and then 3D-printed. Surgeons use this tangible model to perform reduction and then to pre-contour the plate [13,14,15,19,29,33,41];

Virtual planning and reduction and 3D-printed model based approach: 3D printing a plastic plate after virtual fracture reduction and then pre-contouring the metal fixation plate based on the plastic plate [30,31]; Virtual reduction of fracture followed by 3D-printed the reduced fracture model and pre-contouring the plate based on this model [23,24,26,29,32,34,41,42]. FDM is the manufacturing process most used in the reviewed studies (18 papers), other reported processes being stereolithography (SLA, DLP), SLS or MJ.

In 17 reviewed papers Mimics software (Materialise, Leuven, Belgium) was used for transforming patient CT scanning data (DICOM) into 3D virtual anatomical models and then for saving them in STL file format for the 3D printing process. For the same purpose, open-sources software such as OsiriX (Pixmeo, Geneva, Switzerland), or inVesalius was also used by several researchers in combination with Meshmixer (Autodesk, San Rafael, CA, USA) or Meshlab (Visual Computing Lab, Pisa, Italy) for further processing the 3D virtual anatomical models and preparing them for 3D printing.

### 3.6. Reviewed Studies Timeline

**Figure 3:**
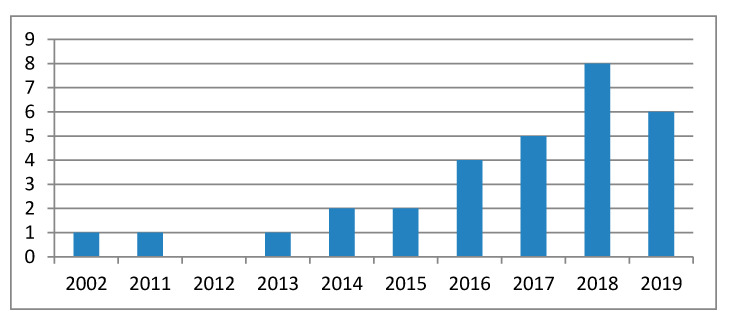
Timeline of included studies (year/no. of studies).

Figure 3 presents a timeline of the included studies. One report was published in 2002 and the next one in 2011. The number of publications steadily increased since 2014–2015. In 2018 almost twice as many papers were published as in 2017. Some of the articles published in 2019 were found at the end of 2018, before being printed. A clear tendency of using the 3D-printed models for orthopedic plate precontouring can be observed in the last couple of years.

## 4. Discussions

All reviewed studies showed common advantages in using patient specific 3D-printed precontouring templates: a decrease in the surgical time and blood loss, improved assessment of fracture configuration and selection of screw and fixation plates based on the existence of a tangible replica of patient bones. It should be noted that applying several bending maneuvers over the plate might increase the risk for plate failure [44], therefore a 3D-printed model becomes a useful tool for a correct plate precontouring without many attempts. Additionally, the reported quality of anatomical reduction is similar to that obtained conventionally. The disadvantages refer mainly to the duration of the virtual modeling process (especially for the approach including virtual reduction) and duration of the 3DP process. However, there are solutions to address the printing time limitation as outlined in the conclusion section. The cost for this type of 3DP medical application was not reported as a shortcoming.

The positive outcomes give an optimistic perspective on the use of this 3DP technology application in orthopedics. This perspective is also related to the increasing number of studies published on this topic in the last couple of years (Figure 3). However, it should be noted that only two studies were identified as belonging to the randomized controlled type. The results of the systematic review showed the predominance of case reports (11 studies) and series of cases (10 studies) followed by retrospective non-randomized series of cases (seven studies) as synthetized in Table 2 and Table 4. As a consequence, more data are needed to correctly assess the clinical feasibility of these 3D-printed contouring templates.

The FDM process is reported as used mostly for manufacturing the precontouring templates, which can be explained based on equipment and material availability and affordability. Eighteen reviewed papers mention using this process, while four other 3DP processes are used in 12 studies.

In ten papers, the researchers used the virtual planning and reduction simulation followed by the 3DP of physical replicas (Figure 2). This is a more complex approach, requiring more modeling time and skills compared to the virtual reconstruction of the injured zone or the virtual reconstruction and mirroring of the healthy zone that is used in 20 (out of 30) revised studies.

The only quantitative data for comparatively evaluating the outcomes, 3DP-assisted procedure vs. classical procedure could be inferred from the non-randomized and randomized cases (Table 4).

### 4.1. 3DP Templating for Acetabular Zone

Most frequently, the use of 3D-printed models for reduction plate precontouring is reported for acetabular fractures (18 out of 30 papers in this study). Several reasons can explain this fact. First, the acetabulum is a particular osseous structure with a complex anatomy, difficult to assess by conventional radiological examination and with limited access to surgical site [30,31]. Then, the acetabulum is the place for complex fracture patterns, with multiple fracture lines and bone fragmentation, which can be easily underestimated on conventional radiologic exam. The complex three-dimensional shape of the bone demands a high understanding of reduction steps, necessary to achieve an anatomical reduction of acetabulum as part of the pelvic ring; this is one reason for the steep learning curve in this surgery type [12,13]. Thus, the advantages offered by a 3D-printed 1:1 replica of patient acetabulum refer to both assessing the fracture position and pattern, and precontouring plates, improving surgery preplanning. Several options and modalities can be used as surgical treatment, plate fixation being a commonly used technique [39]. Contouring the plates during surgery increases the surgical time and could produce imprecise, unreliable results leading to sub-optimal reduction [23]. Studies report the symmetry of the hemipelvis of healthy patients, except for some rotational parameters [45], implying that the replica obtained after a mirroring process is similar to the affected hemipelvis before injury. The physical replica may be used for surgery planning: plate type, length and curvature can be properly chosen by a process of fitting to the 3D print (used as a template). Additionally, correct positioning of the screws (in respect to good bone quality and safe zone of implantation), as well as screw length may be addressed [46]. This determines a consistent reduction of both surgical time (skin to skin) and instrumentation time (time spent to fix the fracture and to accommodate the implant) [13,16]. Blood loss can be better managed when using 3D printing models. Several studies reported consistent reduction of blood loss (e.g., [13,16,33]), while others note the reduction as not significant [30,31]. The accuracy of acetabulum fracture reduction is reported to be similar or slightly better in cases using 3D-printed models for plate contouring (e.g., [20,30,31]).

The treatment for pelvic fracture is also challenging for orthopedic surgeons. For Tile fracture type C this challenge is at its maximum [47]. A full-size 3D-printed model can support the surgeon in better understanding the fracture and selecting the steps in reduction. It also improves the ability to choose the right implant and number, size and good position of screws. All these contribute to shorter operation time, decreased blood loss and blood transfusion [28]. Posterior ring fixation is of paramount importance for fracture management, but anterior ring fixation has its indications [48]. Considering the specific local anatomy with curving planes, fitting a plate to restore it can be a difficult job. 3D-printed models have been reported to support this process [49]. Surgeons can better select the approach, manipulate the fracture fragments and choose the best sequence of reduction and accurately pre-bend the implant. This results in a minimally invasive approach, shorter operation time and decreased blood loss when compared to the conventional technique [24]. When using minimal invasion incision, one may face surgical difficulties during reduction where additional intraoperative plate bending may be needed [32].

### 4.2. 3DP Templating for Clavicle Zone

Clavicle fracture is another area where 3D-printed models for plate precontouring has been used lately, five out of 30 studies in our review being focused on this subject. There are several reasons in favor of this fact: (a) the clavicle is a bone with a particular S shape and many variations, thus fitting an implant is a complex and challenging job; (b) recent studies support the operative treatment for clavicle fracture, but open reduction and osteosynthesis include stripping of periosteum and may result in a delay of union and even non-union [50]; (c) the MIPO (minimally invasive plate osteosynthesis) surgery used in clavicle fracture has proved to have better results [51] and it is easier to perform if a careful plate selection with perfect fitting is done before surgery. In the reviewed studies [22,25,27,37] mirrored models were 3D-printed based on the similarity with the uninjured clavicles [52]. Once the physical replica is available, a simulation of the surgical technique can be done, and the proper plate with the correct length and conformity, as well as proper holes and screw lengths can be set. MIPO technique is salutary in clavicle fracture, but the surgeon may face intraoperative difficulties in choosing the right plate and accommodating it to the bone. The reviewed studies pointed out that 3D printing technology is helpful in this area by saving surgery duration, lowering fluoroscopy use and ensuring better implant conformity. As bilateral CT examination is needed during the process, 3D printing is not an indication of simple fracture cases, but of cases with comminution in which a minimally invasive technique is to be used [27]. The 3D-printed model was also used for cases of mid-shaft clavicle nonunion where correct clavicle length needed to be estimated before surgery [37]. Both clavicle models were 3D-printed and the mirrored one was used to choose the type of plate and its optimal location. For selecting the best plate, two criteria can be taken into account: maximum bone-to-plate contact and a minimum of three screws on medial and lateral side. The models can be obtained on a low budget, using in-hospital 3D printers and may improve surgical time, reduction accuracy and pre-determine the graft need.

### 4.3. 3DP Templating for Calcaneus Fractures

Displaced calcaneus fractures were also considered in two reviewed articles [21,39]. These fractures pose difficulties both in terms of selecting appropriate treatment and complication rate. For surgical treatment an adequate surgical exposure is needed. For this reason, lateral extensile approach is still mostly used. Several complications are related to it, leading to a total of about 37% incidence rate of complications [53]: postoperative wound infection rate, reported between 2% and 25% of cases [54], soft tissue necrosis and wound healing delay. The particular anatomy of calcaneus, with multiple articular surfaces and nervous structure vicinity makes the approach difficult. Limited exposure, as in sinus tarsi or sub-talar approach, is presumed to lower the rate of wound complications. However, limited exposure surgery may face difficulties and here 3D printing models can be helpful as proved by the surveyed literature. A 3D-printed model obtained with mirroring technique is an accurate replica of the injured calcaneus. It can be used to establish the optimal screw trajectory and to pre-shape the plate [39]. In this manner the reduction of fracture can be better judged during surgery by perfectly fitting the plate to the bone. The 3D-printed replica can also be used in educating surgeons not familiarized with calcaneus pathology and for real-size fracture pattern understanding [21].

### 4.4. 3DP Templating for Chest Zone

3D-printed models have also been used in chest wall trauma, especially in cases with multiple rib fractures complicated with flail chest (2 papers out of 30). Open reduction and internal fixation may be recommended for decreasing mortality, reducing time of patient mechanical ventilation and decreasing patient hospitalization [55]. Rib fixation is generally achieved with plates, and for a perfect fitting, they should be bent prior to fixation according to fracture pattern and patient specific anatomy Moreover, the rib thickness can be properly evaluated and the screw lengths precisely determined [34] when a tangible replica is available. In this approach, the skin incision can be decreased, a limited exposure of the rib can be used, surgical and general anesthesia time is considerably reduced, blood loss is also diminished, complex fracture can be assessed and reduced more easily, as well as fractures posteriorly located.

### 4.5. 3DP Templating for Humeral Zone

Significant shortened surgical time and decreased blood loss were reported for proximal third humeral shaft fracture in elderly patients where 3D models were used to pre-bent the helical plate during a minimally invasive technique [38].The physical model allowed an accurate preoperative bending according to the patient’ specific anatomy, with no need for supplementary adjusting during surgery. Additionally, a precise fracture pattern understanding and plate bending and location made the procedure easier even for less-experienced surgeons.

Infrequent pathology as os-acromiale and acromion fractures can also benefit by 3D printing technology. Besides the fact that acromion fracture is uncommon, there is a high inter-individual variability in its shape [56]. The 3D-printed model enhances surgeon ability to choose the right implant among the available clavicle plates, and the proper location to achieve good fixation [15]. It can offer a good alternative to other fixation techniques as cannulated screws or tension band technique, especially in cases with small fragments. It also enables the surgeon to have a better understanding of fracture pattern and underlining pathology in both visual and tactile approach. Therefore, an adequate approach can be selected and potential difficulties related to surgery can be judged in advance. As the surgeon will have a better understanding of the situation, he will need to measure less, and a shorter surgical and general anesthesia time will be achieved. Reported results are good for acromion fracture and more variable for os-acromiale [15].

### 4.6. 3DP Templating for Scaphoid Zone

For small- dimension bones as scaphoid, correct diagnosis in fracture or non-union cases can be difficult on plain x-ray investigation. Therefore, CT examination is usually recommended [57]. The use of 3D-printed models has been reported also in scaphoid non-union cases as training tool [35]. Surgeons simulated surgery on synthetic 3D-printed bones that were also used for pre-shaping the plate, and then for evaluating the graft amount needed during surgery. Altogether with the previously presented benefits, the authors mentioned the need for bilateral CT examination and corresponding additional costs; similarly, one should have in mind that the delay of surgery is not significant when not dealing with an acute case.

### 4.7. 3D Printing Process Related Aspects: Manufacturing Time and Costs, Advantages and Disadvantages

Reviewed studies mentioned that because of the relatively long development times for 3D prints (especially when these steps are outsourced and not performed within hospitals) these models are not suitable for emergency situations. However, in the revised cases the surgical intervention was not recommended immediately, the time for preparing 3D prints causing additional and undesirable delays [21].

In general, the list of disadvantages is focused on aspects related to the 3D printing process: manufacturing time and cost (Table 1). Here it should be noted that a cost of 12 euros [17,18] refers only to the material cost. If the manufacturing process takes place outside the clinic/hospital, at 3D printing services providers, the reported costs and delivery times are relatively high. Kim et al. [27] reported 2–3 days for the entire process (CT scanning, anatomical reconstruction, 3D printing, sterilization and delivery). Therefore, Belien et al. [15] recommended ‘in-house’ 3D printing for circumventing some of the additional costs. The reported 3D printing time for large models such as the pelvis is up to 10h [29]. For reducing this time, setting a smaller infill and optimizing building orientation could represent solutions. However these solutions cannot save more than 2–3 h. Another solution is to 3D print only parts of the anatomical models, for instance only the injured hemipelvis or the mirrored uninjured hemipelvis depending on the approach. The type of 3DP process can also influence the build time of the models and their accuracy. In this sense, Msallem et al. [58] performed a comparative analysis of five AM processes in terms of mandibular replicas dimensional accuracy, the manufacturing time being also noted. Although in the build time evaluation, making comparisons across processes is difficult (for instance, the process parameters settings such as layer height, infill, number of shells, etc. play an important role), it is worth mentioning that in the mentioned research, the FDM models printing times were the smallest and MJ models printing times were the longest.

Another disadvantage mentioned in several papers referred to the time required to prepare the 3D virtual models for 3D printing. Moreover, this step needs training people [18]. Maini et al. [31] noted 4.3 h mean time for segmenting the acetabular fractures (the virtual medical modeling step), Hung et al. [24] reported up to 90 min, while Wang et al. [38] needed 3 h for the virtual anatomical modeling. In all these studies, virtual planning and reduction were performed on fractures and this requires more modeling work, dedicated software and specialized knowledge. In studies where the 3D printing model was based on the uninjured side, the modeling steps for reconstructing the anatomy and mirroring the healthy hemi-pelvis, clavicle or ribs took 30 min [21] or as little as 11 min [29].

Potential digital inaccuracies related to the 3D digitally reconstructed anatomical model are mentioned in [34]. Related to this aspect, and based on experience [59] and literature data [60,61,62] we consider that using dedicated medical modeling software operated by specialists can reduce the risk.

As mandatory for the 3DP process is the availability of a 3D virtual anatomical model; this can be obtained by CT scanning and implies a radiation doze for the patient. This aspect is noted by Kim et al. [27] who mentioned that CT scans are not a practice for clavicle fractures or by Chana-Rodríguez et al. [18]. However, the reduction of surgery time in which x-ray/fluoroscopy is used intra-operatively can bring some compensation from this point of view.

## 5. Conclusions and Future Perspectives

3D-printed anatomical models used as templates for precontouring fixation plates in orthopedic surgeries were reported for various complex fractures (acetabulum, pelvis, clavicle, chest wall, calcaneus, humeral shaft, acromion), as well as in non-union cases (scaphoid) and corrective osteotomies. By far, these 3D-printed models were mostly used as aids in acetabular fracture surgeries.

The review indicated an increase in the use of such models over the last couple of years and a positive impact on the surgical technique and on the training of less-experienced surgeons. However, only few studies compare the new techniques assisted by 3DP with conventional surgery.

Advantages such as a significant decrease in surgical time and blood loss reduction were reported, along with a better understanding of fracture patterns which is usually associated to a tangible replica of patient bones. The data gathered for investigating manufacturing time and costs for 3D-printed templating for plates precontouring showed that: the use of low-cost 3D printers provides good results, significantly impacting the costs of the models, but the long development times (virtual modeling stages and 3DP) do not currently recommend this approach for emergencies.

Two approaches are used for developing 3D-printed anatomical models for precontouring fixation plates: medical reconstruction, virtual planning and fracture reduction followed by 3D printing the model; medical reconstruction followed by 3D printing the model of the mirrored uninjured side. Knowledge of virtual medical reconstruction, virtual medical planning and simulation is mandatory, this aspect probably hindering the spreading of this approach based on the use of 3D printing technology for fixation plates precontouring.

Besides proving the efficiency and effectiveness of this application by conducting more randomized clinical studies, future research work should also be focused on solving the mentioned shortcomings: reducing the medical reconstruction time by automating this process (for instance, with the use of Artificial Intelligence-based solutions for 3D modeling from patient scanning) and reducing the manufacturing time by automatically dividing models into smaller parts/batches and simultaneously constructing 3DPs on several printers. Moreover, as mentioned in the discussion section, further 3DP time reduction can be obtained by reducing the infill percentage. However, this comes with a decrease in the mechanical resistance of the template which can be avoided if adaptive infill is used.

## Figures and Tables

**Table 1:** Details of studies included in the systematic review.

Reference	Type of Intervention	No. of Patients Treated Using 3D Prints	Design Study, Level of Evidence	Approach	Software for 3D Model	3D Printing (Printer, Material, Time, Cost)
Bagaria et al. [13] Year: 2011 Country: India, China	Acetabular fracture, femoral condyle fracture, calcaneal fracture	4	Case series LOE-IV	3DP model of fractured acetabulum (with indelible ink marked zones of fracture) used as template for precontouring the plate	Mimics (Materialise, Leuven, Belgium)	FDM process, ABS 19 euros
Battiato et al. [14] Year: 2017 Country: Italy	Both acetabular fractures	1	Case report LOE-V	3DP of entire pelvis used for fractures reduction and then for precontouring the plates	Mimics (Materialise, Leuven, Belgium)	SLS process, Polyamide PA2200, 46 h the entire development process
Belien et al. [15] Year: 2017 Country: Belgium	Os acromiale, acromial fracture	5	Case series LOE-IV	3DP model of fractured bone after fracture reduction used as template for precontouring the plate	in Vesalius (CTI, SP, Brazil), Rhinoceros 5 (Robert McNeel & Associates, Seattle, USA), Meshmixer (Autodesk, San Rafael, USA), Netfabb Pro (Autodesk, San Rafael, USA)	Makerbot Replicator 2, FDM process
Brown et al. [16] Year: 2002 Country: USA	Acetabular fracture	8	Case series LOE-IV	3DP model of the mirrored model of uninjured acetabulum for precontouring the plate	Mimics (Materialise, Leuven, Belgium)	Actua 2100, SLA, Wax, then mould methyl methacrylate
Chana-Rodriquez et al. [17] Year: 2016 Country: Spain	Acetabular fracture	1	Case report LOE-V	3DP model of the mirrored model of uninjured acetabulum used as template for precontouring the plate	OsiriX (Pixmeo, Geneva, Switzerland), Meshlab V1.3.3 (Visual Computing Lab, Pisa, Italy), Meshmixer 2.4 (Autodesk, San Rafael, USA	Da Vinci 3D printer, FDM process, ABS, 11 h, 12 euros
Chana- Rodriquez et al. [18] Year: 2018 Country: Spain	Acetabular fractures	20	Prospective case series LOE-IV	3DP model of the mirrored model of uninjured acetabulum used as template for precontouring the plate	OsiriX (Pixmeo, Geneva, Switzerland), Meshmixer (Autodesk, San Rafael, USA) Mean of 10.7 min for medical modeling	Da Vinci 3D printer, FDM process, ABS Mean of 385 min for 3D prints manufacturing 12 euros
Chen YY et al. [19] Year: 2018 Country: China	Rib fractures	16	Retrospective review 48 patients LOE-IV	3DP model of fractured ribs/rib (with indelible ink marked zones of fractures) used as template for precontouring the plate	-	UP-BOX 3D Printer, FDM process, ABS 5–6 h for 3DP printing
Chen K et al. [20] Year 2019 Country: China	Bicolumnar acetabular fracture	28	Retrospective analysis, 52 patients, LOE-III	Virtual fracture reduction + 3DP reduced fracture model for precontouring the plate	Mimics 16.0 (Materialise, Leuven, Belgium)	PLA, 36 h total time (including sterilization, bending), 65 euro
Chung et al. [21] Year: 2014 Country: S. Korea	Calcaneal fracture	1	Case report LOE-V	3DP model of contralateral uninjured calcaneus used as template for precontouring the plate	Mimics (Materialise, Leuven, Belgium), 30 min modeling	Probably FDM process 3 h printing
Hao et al. [22] Year: 2019 Country: China	Midshaft clavicle fracture	1	Case report LOE-V	3DP model of contralateral uninjured clavicle used as template for precontouring plate	Mimics 17.0 (Materialise, Leuven, Belgium)	DLP process
Hsu et al. [23] Year: 2019 Country: China	Acetabular fracture	12	Retrospective study with control group LOE-III Total of 29 patients	Virtual reduction of fracture + 3DP model of reduced fracture used as template for precontouring plate	Mimics 19.0 (Materialise, Leuven, Belgium)	Up Box+, FDM process
Hung et al. [24] Year: 2019 Country: China	Pelvic ring fractures	16	Retrospective study, non-randomized with control group LOE-III Total of 30 patients	Virtual reduction of fracture + 3DP model of reduced fracture used as template for precontouring the plate	Mimics 19.0 (Materialise, Leuven, Belgium) Up to 90 min virtual reduction for complex cases	UP BOX+ 3D printer, FDM process Less than 24 h the entire process, 20 euros
Jeong et al. [25] Year: 2014 Country: S. Korea	Clavicle shaft fracture	1	Case report LOE-V	3DP of contralateral clavicle used as template for precontouring the plate	-	$20, 3 h for 3DP, 3 h for plate bending and sterilization
Kataoka et al. [26] Year: 2013 Country: Japan	4 cubitus varus, 1 cubitus valgus, 4 diaphyseal malunions of the forearm	9	Series of cases, LOE-Therapeutic IV	Virtual planning and simulation using contralateral normal bone as template + 3DP of repositioned bone models as template for precontouring the plate	Bone Simulator (Orthree, Osaka, Japan)	Eden 250, Objet, Medical grade resin
Kim et al. [27] Year: 2015 Country: S. Korea, China	Midshaft clavicle fracture	7	Series of cases (technical note) LOE-IV	3DP model of contralateral uninjured clavicle used as template for precontouring the plate	Mimics (Materialise, Leuven, Belgium)	Project x60 series, $100, 2–3 days the whole process CT to solid model
Li L et al. [28] Year: 2017 Country: China	Pelvic fracture	28	Retrospective review (long-term follow up study with control group), Total of 64 patients LOE-III	3DP model of pelvis used for simulating operation and then for precontouring the plates	Mimics 14.0 (Materialise, Leuven, Belgium)	Yinhua Rapid Prototyping 3D printer, probably FDM process
Li YT et al. [29] Year: 2019 Country: China	Hip dislocation combined with acetabular fracture	7	Retrospective review control group Total of 16 patients LOE-III	Virtual reduction by mirroring contralateral, uninjured side + 3DP model of reduced model used as template for precontouring the plate	Mimics 19.0 (Materialise, Leuven, Belgium), 11 min for modeling	Up Box+ 3D printer, FDM process, 10 h for 3D printing
Maini et al. [30] Year: 2018 Country: India	Acetabular fracture	10	Prospective randomized case control study Total of 21 patients LOE-II	3DP model of fractured acetabulum followed by its reduction and its use as template for precontouring the plate	Mimics 8.13 (Materialise, Leuven, Belgium)	Eosint P380, SLS, nylon polyamide, $15–20
Maini et al. [31] Year: 2018 Country: India	Acetabular fracture	12	Randomized case control study 25 patients LOE-II	Virtual planning and simulation for reducing fracture followed by virtual modeling the plate (virtual plating) + 3D-printed plate model as template for precontouring the metal plate	Mimics and 3-Matic (Materialise, Leuven, Belgium), Average time: 4.3 h	FDM, PLA, $4
Nie et al. [32] Year: 2018 Country: China	Pubic rami fractures	30	Consecutive case series LOE-IV	Virtual planning and reduction of fracture + 3DP model of reduced fracture used as template for precontouring plate	Mimics 10.01 (Materialise, Leuven, Belgium)	FDM process, ABS material probably
Shon et al. [33] Year: 2018 Country: S. Korea	Both-column acetabular fractures	5	Series of cases LOE-IV	3DP model of fractured acetabulum (with indelible ink marked fracture line) followed by reduction and fixation with glue, thus reduced model being used as template for precontouring the plate	-	Edison 3D printer, FDM process, PLA, 3 h total development time for the 3D print, $30
Smith et al. [34] Year: 2018 Country: USA	Rib fractures	1	Case report LOE-V	Virtual reduction using mirroring of contralateral uninjured side + 3DP model with reduced fractured and marked fracture lines used as template for precontouring plates	D2P (3D Systems, Rock Hill, USA), Geomagic Freeform Plus (3D Systems, Rock Hill, USA)	ProX 800 3D printer, SLA, ClearView polycarbonate-like resin
ten Berg et al. [35] Year: 2017 Country: The Netherlands	Nonunion of scaphoid fracture	8	Series of cases (short report letter) LOE-V	3DP model of uninjured contralateral bone for plate bending	Custom software (C++(Visual Studio 2005, Microsoft, Redmond, USA), Visualization ToolKit (VTK 5.0.4, Kitware, Inc., NY, USA), Insightt ToolKit (ITK 3.6.0, Kitware, Inc., NY, USA)	Blue printer M2, Selective Heat Sintering, Thermoplastic powder
Upex et al. [36] Year: 2017 Country: France	Acetabular fracture	1	Case report (technical note) LOE-V	3DP model of the healthy hemipelvis used as template for precontouring plate	OsiriX (Pixmeo, Geneva, Switzerland), Meshmixer (Autodesk, San Rafael, USA)	Ultimaker, FDM process, PLA, 6 euros
Van Doremalen et al. [37] Year: 2016 Country: The Netherlands	Midshaft clavicle fracture	1	Case report LOE-V	3DP model of contralateral intact clavicle for plate bending	Matlab(MathWorks, Natick, USA), Meshlab (Visual Computing Lab, Pisa, Italy	BQ Witbox, FDM process, PLA, 4 h total time for the whole process (modeling, 3DP, bending)
Wang et al. [38] Year: 2018 Country: China	Humeral shaft fracture	21	Retrospective review, 46 patients, comparison 3DP model with Synbone model LOE-IV	3DP model of intact bone used as template for precontouring the plate	Mimics 16.0 (Materialise, Leuven, Belgium) 3 h modeling time	Lite, RS6000, DLP process, ultraviolet curable resin
Yao et al. [39] Year: 2019 Country: China, Australia	Calcaneal fractures	25	Case series LOE-IV	3DP model of uninjured calcaneus used as template for precontouring plate	Mimics 15.0 (Materialise, Leuven, Belgium)	Makerbot Replicator 3D printer
Yu et al. [40] Year: 2015 Country: UK	Both column acetabulum fractures	2	Cases report LOE-V	3DP model of contralateral uninjured side used as template for precontouring the plate	-	Objet Eden 250 3D printer, SLS process, MED610 polymer
Zeng et al. [41] Year: 2016 Country: China	Acetabular fracture	10	Series of cases LOE-IV	Virtual fracture reduction + 3DP reduced fracture model for precontouring the plate	Mimics 14.0 (Materialise, Leuven, Belgium)	Makerbot Replicator 2, FDM process
Zhuang et al. [42] Year: 2016 Country: China	Acetabular fractures (7 fractures anterior column, 4 anterior column with posterior hemitransverse, 1 anterior column with the pubic symphysis)	12	Case series LOE-IV	3D printed model on uninjured hemipelvis with marked fracture lines used as template for precontouring the plate	-	Mira ProJet 3510 3D printer, MJ process, ultraviolet curable resin

**Table 2:** Synthesis of studies design included in review.

Studies Design	Case Reports	Series of Cases	Non-Randomized Clinical Studies	Randomized-Clinical Studies
Representing % from the total No. of papers	36.67%	33.33%	23.33%	6.67%
No. of patients in 3DP group	10	143	128	22
No. of patients in control group	-	-	233	24

**Table 3:** Synthesis of anatomical locations considered in the included studies.

Anatomical Location	Total No. of Patients Using 3D Prints	Total No. of Studies Per Anatomical Location	Total No. of Patients in Studies
Acetabulum	206	18 (60%)	415 (206 3DP + 209 control)
Clavicle	15	5 (17%)	15
Rib	17	2 (6.8%)	65 (17 3DP + 48 control)
Humerus	21	1	21
Cubitus	9	1	9
Scaphoid	8	1	8
Calcaneus	27	2	27

**Table 4:** Data on reported advantages of using 3DP for plate precontouring.

Study	Mean Blood Loss (mL)		Mean Surgical Time (min)/Instrumentation Time (Min)		Quality of Reduction
	3DP Group	Conventional Group	3DP Group	Conventional Group	
Chen YY et al. [19]	-	-	125 ± 33.44	175.24 ± 60.58	-
Chen K et al. [20]	696.0 7 ± 66.54	833.75 ± 227.44	157.5 ± 20.48	187.08 ± 35.81	Similar
Hsu et al. [23]	433.33 ± 317.28	958.33 ± 427.10	199.00 ± 50.29	274.17 ± 80.95	Similar
Hung et al. [24]	275.00 ± 196.64	549.29 ± 404.43	206.13 ± 70.32/ 45.63 ± 15.26	276.21 ± 89.53/ 102.86 ± 25.85	Similar
Li L et al. [28]	481.4 ± 103.2	771.1 ± 114.4	128.9 ± 59.2	191.4 ± 85.1	Better in 3DP group (Matta score)
Li YT et al. [29]	735.71 ± 614.22	742.22 ± 228.68	211.71 ± 52.23/ 38.43 ± 10.81	254.44 ± 34.46/ 71.78 ± 9.69	Similar
Maini et al. [30]	620 ± 246.9	720 ± 286.2	120 ± 37.7	132 ± 41.0	Better in 3DP group (Matta score)
Maini et al. [31]	467	525	111	119	Better in 3DP group
Wang et al. [38]	105.19 ± 14.67	120.80 ± 10.61	42.62 ± 7.61	60.36 ± 10.20	Similar
